# A Comparative Evaluation of Antifungal and Physical Properties When Nanoparticles Are Incorporated Into the Tissue Conditioner: An In Vitro Study

**DOI:** 10.7759/cureus.67348

**Published:** 2024-08-20

**Authors:** S Lavanya, Shafath Ahmed, Vidyashree V Nandini, Abinaya Saravanan, Sadhana KR, Manjula G

**Affiliations:** 1 Prosthodontics, SRM Kattankulathur Dental College and Hospital (SRMIST), Chengalpattu, IND

**Keywords:** antifungal, candida albicans, surface roughness, tissue conditioner, nanoparticle

## Abstract

Objective

The objective of this in vitro study was to comparatively evaluate the antifungal and physical properties of tissue conditioner incorporated with nanoparticles (NPs) of different types and concentrations.

Materials and methods

A total of 198 tissue conditioner samples were used in this study. The samples were categorized into a control group, namely, tissue conditioner without NPs (Group 1), and test groups, namely, tissue conditioner incorporated with zinc oxide (ZnO) NPs (Group 2) and magnesium oxide (MgO) NPs (Group 3). The antifungal properties and surface roughness of the samples were evaluated. The groups were further subdivided into seven subgroups: control (without NPs), 5% ZnO NPs, 10% ZnO NPs, 15% ZnO NPs, 3% MgO NPs, 5% MgO NPs, and 7% MgO NPs by weight. Surface roughness was measured using an optical profilometer, and antifungal activity was measured in terms of the diameter of the inhibition zone (DIZ) using the well diffusion method over seven days.

Results

The result showed that the 5% ZnO NPs subgroup had the lowest mean surface roughness, whereas the 15% ZnO NPs subgroup had the highest antifungal activity. Increasing the concentration of NPs increased the antifungal property, and there was a steady decrease in DIZ from day one to day seven in all test groups.

Conclusion

Our results showed that the incorporation of various concentrations of ZnO and MgO NPs into tissue conditioner samples positively affected their physical and antifungal properties. The highest antifungal activity was found in the 15% ZnO NPs subgroup, and the lowest surface roughness was found in the 5% ZnO NPs subgroup.

## Introduction

Complete or partial edentulism is one of the most prevalent dental conditions, which requires the replacement of lost teeth in a way that harmonizes with the orofacial structure. An important part of a complete denture treatment is maintaining the health of the mucosa covering the residual alveolar ridge. Tissue conditioners are polymer-based resin materials applied to the tissue side of the denture base. These materials help to reduce the force per unit area delivered to the supporting tissues by allowing greater dispersion of forces [1].

A tissue conditioner absorbs the forces generated by chewing and serves as a buffer between the denture’s intaglio surface and the underlying oral tissues, providing a cushioning impact and aiding in the healing of the inflamed mucosa [2,3]. Chemical-based resins are of two types: heat-activated and chemically activated. These polymers are delivered as powders, which are then combined with liquids containing 60% to 80% of a plasticizer. Usually, a large molecular particle such as dibutyl phthalate serves as the plasticizer. Although plasticizers increase flexibility, there are drawbacks as well. Plasticizers have the potential to “leach out” of the tissue conditioner, as they do not bond well to the resin mass; as a result, the tissue conditioner becomes rigid [2]. Nanotechnology has revolutionized the field of dentistry by enabling the manipulation of matter at the molecular level [4,5].

Nanoscale materials exhibit unique characteristics due to their increased surface area, advanced fabrication methods, and ability to modify specific physical and chemical properties. Their high surface-to-volume ratio enhances their reactivity and catalytic efficiency, making them ideal candidate materials for various applications [6-8].

Zinc, an essential trace element found in hard tissues such as teeth, muscles, bones, and skin, plays vital roles in various physiological processes [2,6]. Zinc oxide (ZnO) can interact with the SH group of enzymes in microorganisms, causing protein denaturation and damage to DNA. 

The antimicrobial properties of magnesium oxide (MgO) have raised interest in its potential application in medicine; its efficacy extends to combatting various microorganisms, including cariogenic oral bacteria such as species of *Staphylococcus mutans* [8].

The aim of the study was to comparatively evaluate the physical and antifungal properties of the tissue conditioner incorporated with the ZnO NPs and MgO NPs. The study began with a null hypothesis that surface roughness and antifungal activity in the tissue conditioner incorporated with ZnO and MgO NPs would be the same.

## Materials and methods

This in vitro study was conducted at SRM Kattankulathur Dental College and Hospital, Chennai, India. The study was approved by the Institutional Scientific and Ethical Review Board of SRM Kattankulathur Dental College and Hospital before its commencement (approval number E268/IEC/2022). The sample size was estimated using G*Power 3.1.9.2 (Heinrich Heine University of Düsseldorf, Germany) software according to a statistical power of 90%.

Synthesis of zinc oxide nanoparticles

ZnO NPs were synthesized using the sol-gel method. First, 8 g of sodium hydroxide and 2 g of zinc acetate dihydrate were dissolved in 40 mL of distilled water to form a sol. The resulting solution was continuously stirred for 30 minutes using a magnetic stirrer. After a few minutes, a zinc hydroxide precipitate formed in the beaker. The precipitate was filtered and rinsed four times with ethanol. The obtained precipitate was then centrifuged for five minutes at 5000 rpm and subsequently dried at room temperature. The sample was placed in a hot-air oven at 1000°C for 24 hours [9,10].

Synthesis of magnesium oxide nanoparticles

Magnesium sulfate was used along with sodium hydroxide to synthesize MgO NPs. First, 0.2 mg of magnesium sulfate was dissolved in 100 mL of distilled water using the standard experimental protocol. With continuous stirring, sodium hydroxide pellets weighing 1 g were added dropwise to the magnesium sulfate solution. The mixture was continuously stirred with a magnetic stirrer for 30 minutes. Then, the mixture was centrifuged for 5 minutes at 5000 rpm and allowed to dry at room temperature. The sample was then placed in a hot-air oven at 1000°C for 24 hours, after which MgO NPs were formed [11].

Scanning electron microscopy analysis

**Figure 1:**
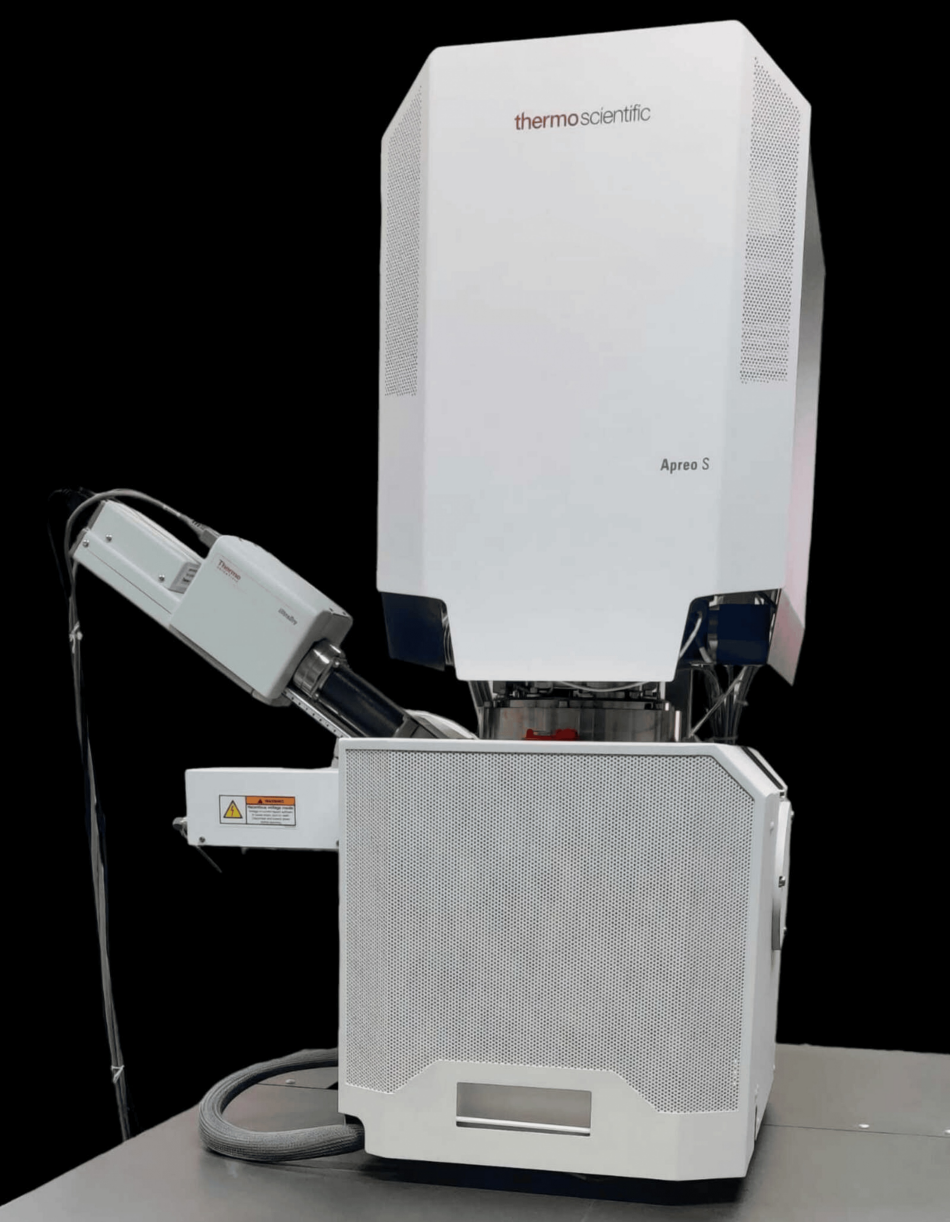
SEM SEM: scanning electron microscope

Scanning electron microscopy (SEM) (Apreo S, Thermo Fisher Scientific India Pvt. Ltd., Mumbai, India) was used to analyze the morphology of the pure ZnO NPs and MgO NPs produced (Figure 1).

**Figure 2:**
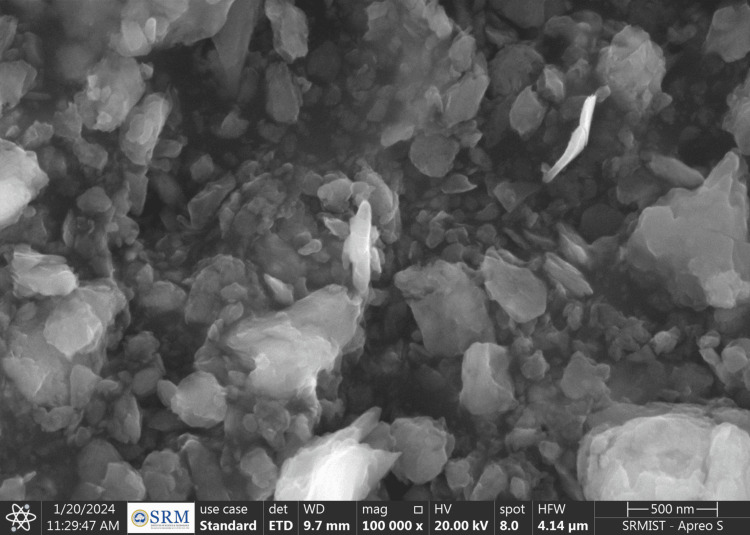
SEM image of ZnO NPs SEM: scanning electron microscopy; ZnO NPs: zinc oxide nanoparticles

**Figure 3:**
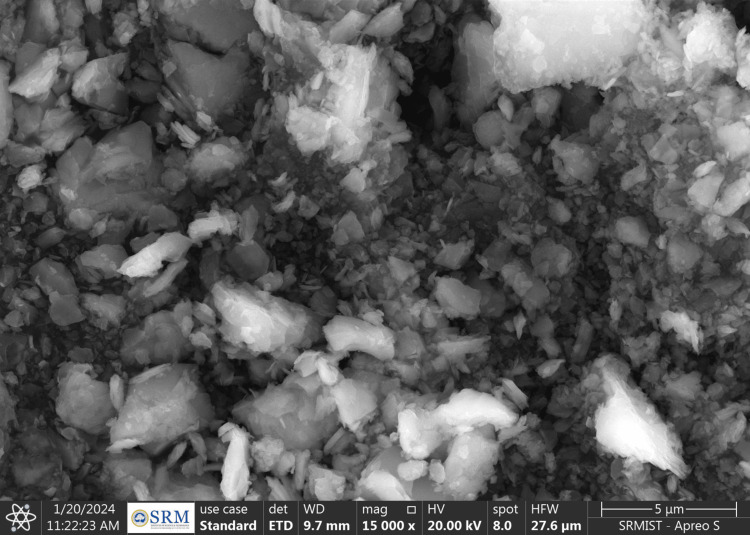
SEM image of MgO NPs SEM: scanning electron microscopy; Mgo NPs: magnesium oxide nanoparticles

The SEM images revealed that the ZnO NPs were agglomerated and arranged in flocks, with an average NP size of <100 nm (Figure 2) [9]. In contrast, the MgO NPs were spherical, with an average NP size of 20-50 nm (Figure 3).

Fabrication of samples

In the current study, tissue conditioner (GC Corporation, Tokyo, Japan) was used in the fabrication of samples. A total of 198 samples were taken, comprising 66 control samples of only tissue conditioner, 66 samples of tissue conditioner incorporated with ZnO NPs, and 66 samples of tissue conditioner incorporated with MgO NPs. The ZnO NP and MgO NP groups were each further divided into three subgroups: 5%, 10%, and 15% of ZnO NPs by weight; and 3%, 5%, and 7% of MgO NPs by weight, with 22 samples in each subgroup. 11 samples from each subgroup were taken for surface roughness testing. These were disc-shaped samples with a thickness of 2 mm and a diameter of 10 mm. Similarly, 11 samples were included from each subgroup for antifungal activity testing [12].

Evaluation of surface roughness

Surface roughness analysis was conducted utilizing the Mitutoyo analog roughness tester (i.e., profilometer) (Bombay Tools Center Bombay Pvt. Ltd., Mumbai). Three measurements were taken for each specimen: one at the central point and two that were 1 mm away from the central point. The average of these three measurements was considered and recorded in the units of μm. For analysis, the square root of the average of the squared deviations of the scan’s departures from the mean line or the average roughness (Ra) parameter was used [13].

Evaluation of antifungal property

**Figure 4:**
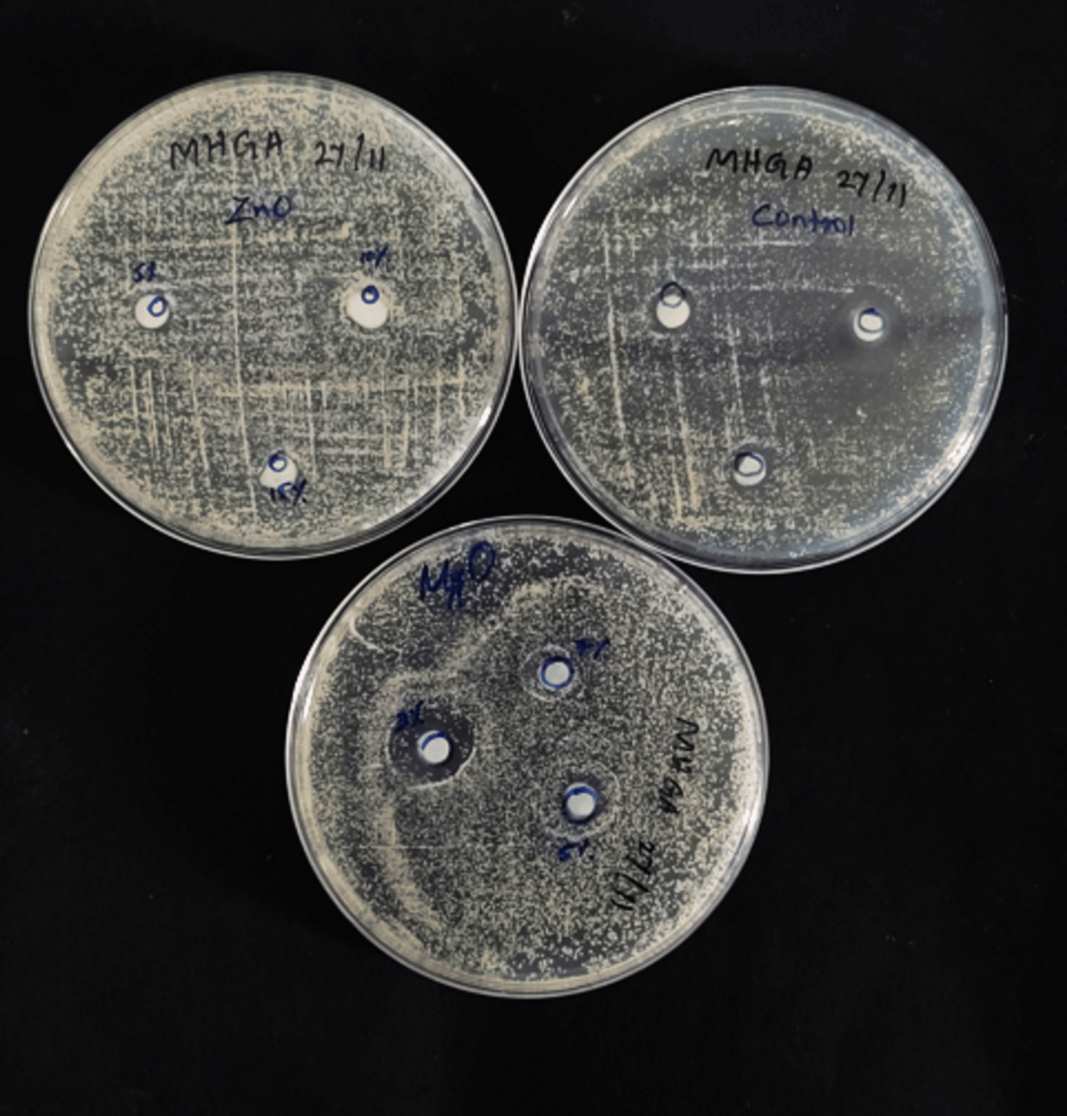
Samples for antifungal property

The antifungal susceptibility of the samples was assessed using the good diffusion method, where 11 samples were taken from each subgroup of the ZnO NPs and MgO NPs groups. A sample of *Candida albicans* (strain 90028 from the American Type Culture Collection) was obtained, subcultured on Sabouraud dextrose agar (SDA) in test tubes, and then incubated at 37°C for 24 hours. After incubation, a *Candida albicans *suspension was prepared by combining *Candida albicans* with sterile saline to achieve a density of 0.5 McFarland, thereby standardizing the concentration. Petri plates with 10 mL of SDA were spread with the activated strains of 100 μL inoculum (1 x 108 cfu/mL) of *Candida albicans* using a sterile swab. Sterile punches were made to hold the tissue conditioner specimens of the three groups and their subgroups (control; 3%, 5%, and 7% MgO; and 5%, 10%, and 15% ZnO) (Figure 4).

**Figure 5:**
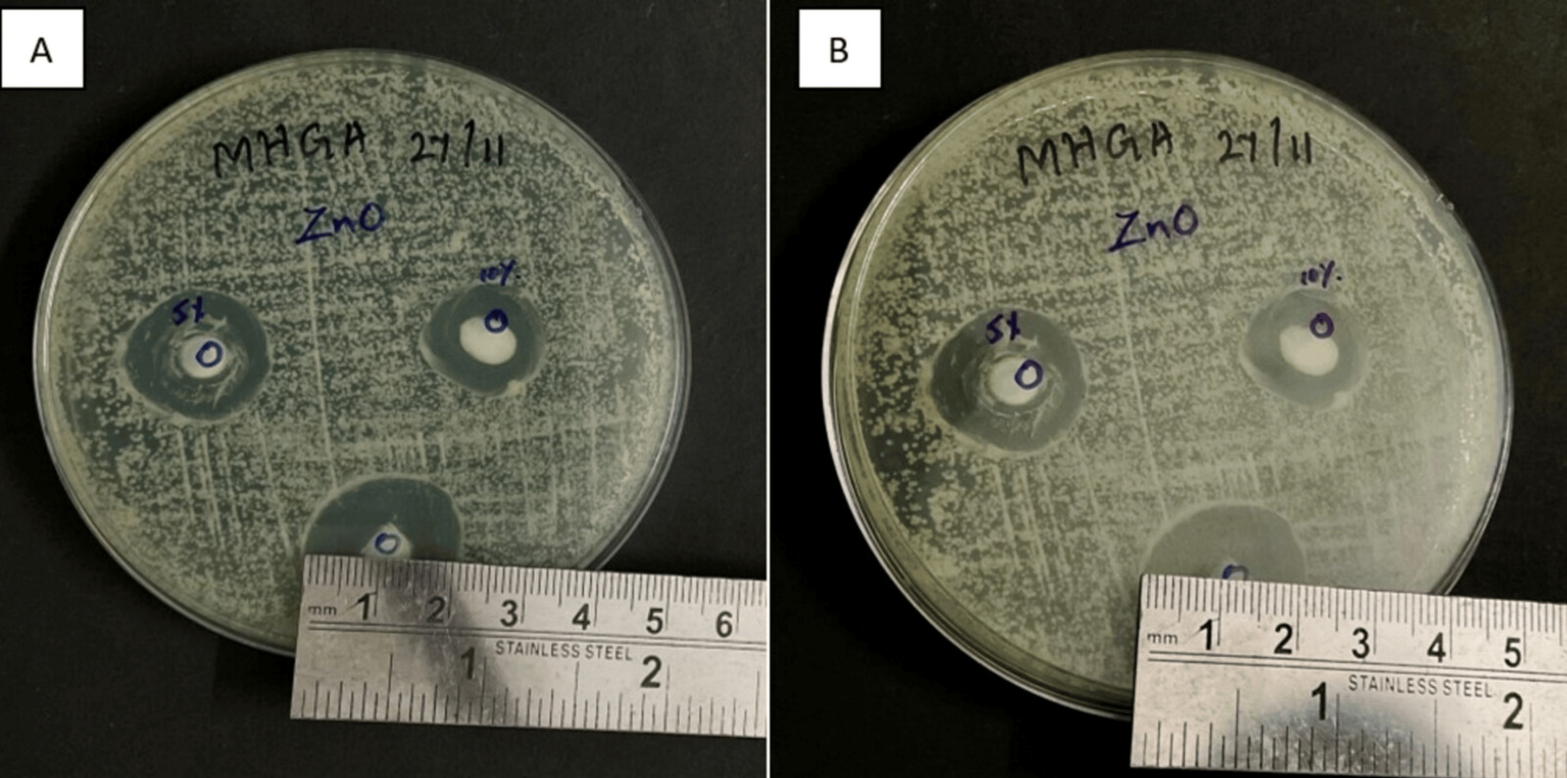
DIZ of ZnO NP sample group. A) On day one and B) on day seven DIZ: diameter of the inhibition zone; ZnO NP: zinc oxide nanoparticle

**Figure 6:**
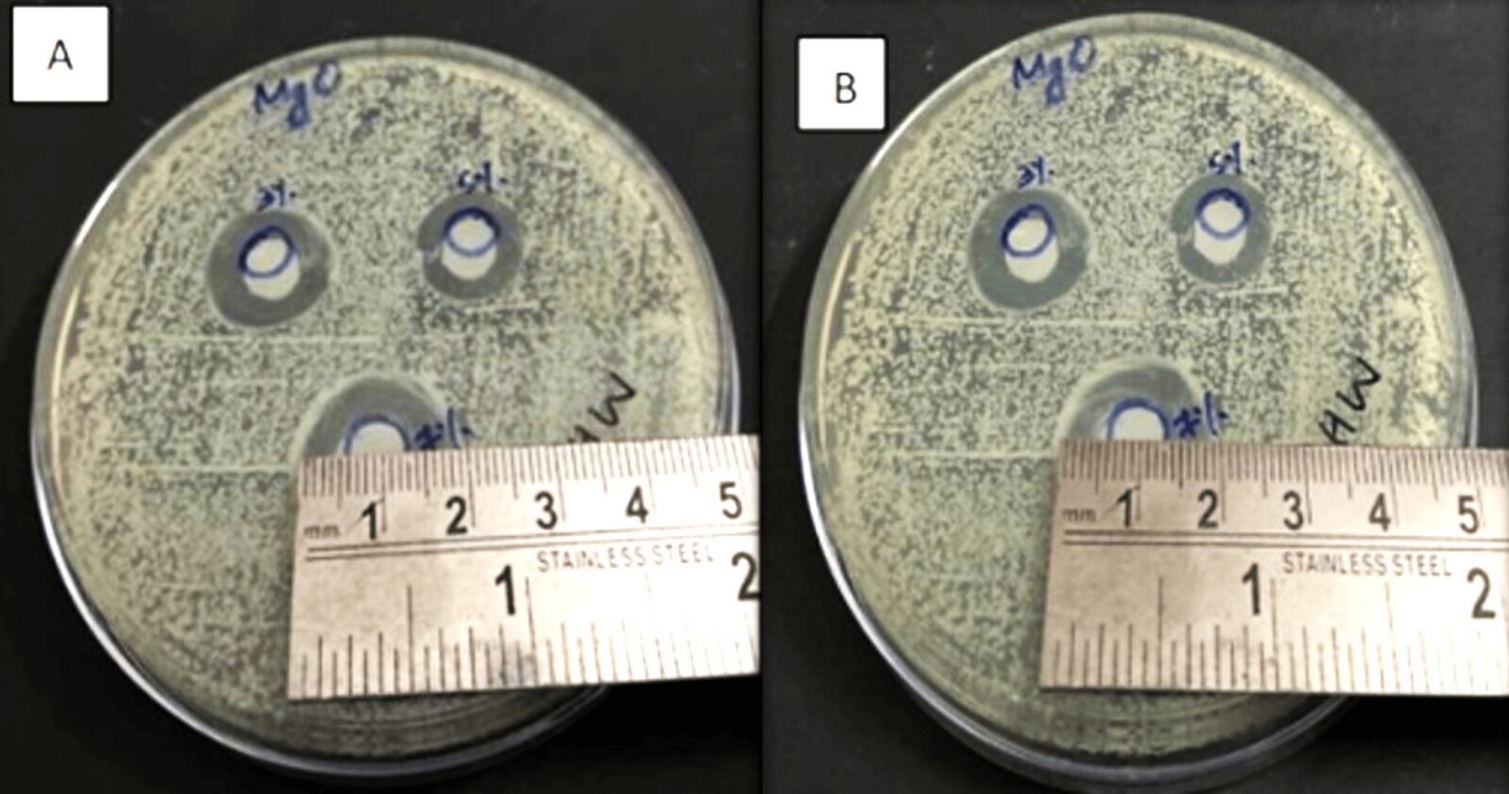
MgO NP sample A) on day one and B) on day seven MgO NP: magnesium oxide nanoparticle

The plates were incubated for seven days at 37°C. On the first and seventh days, the diameter of the inhibition zone (DIZ) was measured using a metallic scale (Figure 5, 6) [14].

The surface roughness and DIZ at days one and seven of the three groups were analyzed using one-way analysis of variance (ANOVA) followed by pairwise multiple comparisons with Tukey’s honestly significant difference test (α=0.05). 

## Results

The lowest mean surface roughness value was observed in the 5% ZnO NPs subgroup (0.731), followed by the 10% ZnO NPs (1.323), 3% MgO NPs (1.547), and 15% ZnO NPs (1.950) subgroups. In contrast, the 5% MgO NPs (3.071), control (3.183), and 7% MgO NPs (3.538) subgroups were found to have the highest mean surface roughness (Table 1). One-way ANOVA showed overall differences in mean values between the control and experimental groups, which were found to be statistically significant (p=0.000).

**Table 1:** Ra between the control group, ZnO NPs, and MgO NPs *Statistically significant (p<0.05), F-value indicates one-way ANOVA value. Ra: surface roughness; ZnO NPs: zinc oxide nanoparticles; MgO NPs: magnesium oxide nanoparticles; ANOVA: analysis of variance

Ra		Mean+SD	95% confidence interval for mean			Sum of squares	df	Mean square	F-value (p-value)
			Lower	Upper					
Control (n=33)		3.183+0.459	3.020	3.346	Between groups	93.067	6	15.511	75.546 (0.000)*
ZnO NPs (n=33)	5% wt	0.731+0.127	0.645	0.816					
	10% wt	1.323+0.456	1.016	1.630					
	15%wt	1.950+0.128	1.864	2.036	Within groups	18.889	92	0.205	
MgO NPs (n=33)	3% wt	1.547+0.469	1.231	1.862					
	5% wt	3.071+0.520	2.721	3.420					
	7% wt	3.538+0.693	3.072	4.004	Total	111.956	98		
Total (n=99)		2.412+1.068	2.199	2.625					

Post hoc analysis revealed statistically significant mean differences between the control group and all subgroups of ZnO NPs (p=0.000), as well as between the control group and the 3% MgO NPs subgroup (p=0.00). No statistically significant differences were found between the control group and the 5% (p=0.991) and 7% of MgO NPs (p=0.203) subgroups; the 10% of ZnO NPs and 3% of MgO NPs subgroups(p=0.908); the 15% of ZnO NPs and 3% of MgO NPs subgroups (p=0.368); or the 5% of MgO and 7% of MgO NPs subgroups (p=0.203) (Table 2).

**Table 2:** Post hoc Tukey test for Ra between the control group, ZnO NPs, and MgO NPs *Mean difference is significant at the 0.05 level. ZnO NPs: zinc oxide nanoparticles; MgO NPs: magnesium oxide nanoparticles; Ra: surface roughness

Dependent variable	(I) Groups	(J) Groups	Mean difference (I-J)	P-value	95% confidence interval	
					Lower	Upper
Ra	Control	5% wt ZnO NPs	2.452^*^	.000	1.977	2.928
		10% wt ZnO NPs	1.860^*^	.000	1.384	2.335
		15% wt ZnO NPs	1.233^*^	.000	.757	1.708
		3% wt MgO NPs	1.636^*^	.000	1.161	2.112
		5% wt MgO NPs	112	.991	-.362	.588
		7% wt MgO NPs	-.354	282	-.829	121
	5% wt ZnO NPs	10% wt ZnO NPs	-.592^*^	.043	-1.175	-.010
		15% wt ZnO NPs	-1.219^*^	.000	-1.802	-.637
		3% wt MgO NPs	-.816^*^	.001	-1.398	-.233
		5% wt MgO NPs	-2.340^*^	.000	-2.922	-1.757
		7% wt MgO NPs	-2.807^*^	.000	-3.389	-2.224
	10% wt ZnO NPs	15% wt ZnO NPs	-.627^*^	.026	-1.209	-.044
		3% wt MgO NPs	-.223	.908	-.806	.358
		5% wt MgO NPs	-1.747^*^	.000	-2.329	-1.165
		7% wt MgO NPs	-2.214^*^	.000	-2.796	-1.632
	15% wt ZnO NPs	3% wt MgO NPs	.403	.368	-.178	.986
		5% wt MgO NPs	-1.120^*^	.000	-1.702	-.537
		7% wt MgO NPs	-1.587^*^	.000	-2.169	-1.004
	3% wt MgO NPs	5% wt MgO NPs	-1.523^*^	.000	-2.106	-.941
		7% wt MgO NPs	-1.990^*^	.000	-2.573	-1.408
	5% wt MgO NPs	7% wt MgO NPs	-.467	.203	-1.04	.115

The antifungal activity of the samples was measured in terms of DIZ (mm). On day one, the largest mean DIZ was found in the 15% ZnO NPs (28.90 mm) subgroup, followed by the 10% ZnO NPs (22.64 mm), 7% MgO NPs (17.81 mm), 5% ZnO NPs (16.65 mm), and 5% MgO NPs (13.24 mm) subgroups. The smallest mean DIZ was observed in the 3% MgO NPs subgroup (11.03 mm). However, on day seven, DIZ was largest in the 15% ZnO NPs subgroup (27.53 mm), followed by the 10% ZnO NPs (21.09 mm), 7% MgO NPs (16.80 mm), 5% ZnO NPs (15.80 mm), 5% MgO NPs (12.20 mm), and 3% MgO NPs (9.73 mm) subgroups, with the last subgroup showing the smallest mean DIZ. The control group showed no signs of DIZ on days one or seven (Table 3). 

**Table 3:** Zone of inhibition (mm) between the control group, ZnO NPs, and MgO NPs *Statistically significant (p<0.05), F-value indicates one-way ANOVA. ZnO NPs: zinc oxide nanoparticles; MgO NPs: magnesium oxide nanoparticles; ANOVA: analysis of variance

Zone of inhibition (mm)		Mean+SD	95% confidence interval for mean			Sum of squares	df	Mean square	F-value (p-value)
			Lower	Upper					
Day 1									
Control (n=33)		0.000+0.000	0.000	0.000	Between groups	9771.781	6	1628.630	307.123 (0.000)*
ZnO NPs (n=33)	5% wt	16.654+2.542	14.946	18.362					
	10% wt	22.645+3.385	20.371	24.919					
	5% wt	28.900+4.582	25.821	31.978	Within groups	487.864	92	5.303	
MgO NPs (n=33)	3% wt	11.036+2.233	9.535	12.537					
	5% wt	13.245+1.931	11.947	14.543					
	7% wt	17.818+1.071	17.098	18.537	Total	10259.644	98		
Total (n=99)		12.255+10.231	10.214	14.296					
Day 7									
Control (n=33)		0.000+0.000	0.000	0.000	Between groups	8783.853	6	1463.975	277.658 (0.000)*
ZnO NPs (n=33)	5% wt	15.809+2.647	14.030	17.587					
	10% wt	21.209+3.219	19.046	23.372					
	15% wt	27.536+4.424	24.563	30.509	Within groups	485.078	92	5.273	
MgO NPs (n=33)	3% wt	9.736+2.056	8.354	11.118					
	5% wt	12.200+2.236	10.697	13.702					
	7% wt	16.809+1.522	15.786	17.832	Total	9268.931	98		
Total (n=99)		11.477+9.725	9.538	13.417					

In the 5% ZnO NPs subgroup, DIZ decreased from day one (16.65 mm) to day seven (15.80 mm). An evident decrease in the antifungal activity was observed between day one and day seven in the various wt% of ZnO and MgO NPs subgroups (Table 4). 

**Table 4:** Post hoc Tukey analysis for the zone of inhibition (mm) between the control group, ZnO NPs, and MgO NPs *Mean difference is significant at the 0.05 level. ZnO NPs: zinc oxide nanoparticles; MgO NPs: magnesium oxide nanoparticles

Dependent variable	(I) Groups	(J) Groups	Mean difference (I-J)	P-value	95% Confidence interval	
					Lower	Upper
Zone of inhibition (mm) on day 1	Control	5% wt ZnO NPs	-16.654^*^	.000	-19.071	-14.237
		10% wt ZnO NPs	-22.645^*^	.000	-25.062	-20.228
		15% wt ZnO NPs	-28.900^*^	.000	-31.316	-26.483
		3% wt MgO NPs	-11.036^*^	.000	-13.453	-8.619
		5% wt MgO NPs	-13.245^*^	.000	-15.662	-10.828
		7% wt MgO NPs	-17.818^*^	.000	-20.235	-15.401
	5% wt ZnO NPs	10% wt ZnO NPs	-5.990^*^	.000	-8.951	-3.030
		15% wt ZnO NPs	-12.245^*^	.000	-15.205	-9.285
		3% wt MgO NPs	5.618^*^	.000	2.658	8.578
		5% wt MgO NPs	3.409^*^	.013	.449	6.369
		7% wt MgO NPs	-1.163	.898	-4.123	1.796
	10% wt ZnO NPs	15% wt ZnO NPs	-6.254^*^	.000	-9.214	-3.294
		3% wt MgO NPs	11.609^*^	.000	8.649	14.569
		5% wt MgO NPs	9.400^*^	.000	6.439	12.360
		7% wt MgO NPs	4.827^*^	.000	1.867	7.787
	15% wt ZnO NPs	3% wt MgO NPs	17.863^*^	.000	14.903	20.823
		5% wt MgO NPs	15.654^*^	.000	12.694	18.614
		7% wt MgO NPs	11.081^*^	.000	8.121	14.041
	3% wt MgO NPs	5% wt MgO NPs	-2.209	.280	-5.169	.751
		7% wt MgO NPs	-6.781^*^	.000	-9.741	-3.821
	5% wt MgO NPs	7% wt MgO NPs	-4.572^*^	.000	-7.532	-1.612
Zone of inhibition (mm) on day 7	Control	5% wt ZnO NPs	-15.809^*^	.000	-18.219	-13.399
		10% wt ZnO NPs	-21.209^*^	.000	-23.619	-18.799
		15% wt ZnO NPs	-27.536^*^	.000	-29.946	-25.126
		3% wt MgO NPs	-9.736^*^	.000	-12.146	-7.326
		5% wt MgO NPs	-12.200^*^	.000	-14.610	-9.790
		7% wt MgO NPs	-16.809^*^	.000	-19.219	-14.399
	5% wt ZnO NPs	10% wt ZnO NPs	-5.400^*^	.000	-8.351	-2.448
		15% wt ZnO NPs	-11.727^*^	.000	-14.678	-8.775
		3% wt MgO NPs	6.072^*^	.000	3.121	9.024
		5% wt MgO NPs	3.609^*^	.007	.657	6.560
		7% wt MgO NPs	-1.000	.948	-3.951	1.951
	10% wt ZnO NPs	15% wt ZnO NPs	-6.327^*^	.000	-9.278	-3.375
		3% wt MgO NPs	11.472^*^	.000	8.521	14.424
		5% wt MgO NPs	9.009^*^	.000	6.057	11.960
		7% wt MgO NPs	4.400^*^	.000	1.448	7.351
	15% wt ZnO NPs	3% wt MgO NPs	17.800^*^	.000	14.848	20.751
		5% wt MgO NPs	15.336^*^	.000	12.384	18.288
		7% wt MgO NPs	10.727^*^	.000	7.775	13.678
	3% wt MgO NPs	5% wt MgO NPs	-2.463	.166	-5.415	.488
		7% wt MgO NPs	-7.072^*^	.000	-10.024	-4.121
	5% wt MgO NPs	7% wt MgO NPs	-4.609^*^	.000	-7.560	-1.657

## Discussion

In our in vitro study, tissue conditioner material used for dentures was comparatively evaluated when it was incorporated with various concentrations of ZnO and MgO NPs. Tissue conditioner is a polymer-based material applied to the tissue side of the denture base. The major drawback of employing a tissue conditioner is that it must be used for only a short amount of time due to the leaching out of plasticizing agents and ethyl alcohol. Therefore, fresh tissue conditioners must be replaced every three to four days. A delay in replacing tissue conditioners causes surface roughness in the denture base and increases the adherence of microbes [2]. ZnO NPs and MgO NPs have obtained significant attention in various fields due to their unique physical and chemical properties. By incorporating ZnO NPs and MgO NPs, it is possible to improve the physical properties, antimicrobial properties, and overall performance of the tissue conditioner [3,9].

According to Verran J, rough surfaces have higher levels of fungal adherence than smooth ones, with rough silicone surfaces harboring more cells than rough acrylic surfaces [15]. A study by Radford et al. found that *Candida albicans* adhered to tissue conditioner materials more frequently than to acrylic surfaces [16]. It has been observed that the addition of nanoscale metal oxides such as ZnO NPs can cause antimicrobial and physical changes in the tissue conditioner [17].

In our in vitro study, ZnO and MgO NPs were synthesized using the sol-gel method. Synthesis of NPs using the sol-gel method allows for the generation of NPs at reduced temperatures, which presents many advantages such as the incorporation of antimicrobial properties and good adaptability [17,18].

Based on the statistical analysis in our study, the lowest surface roughness was found in the 5% ZnO NPs subgroup, and the highest surface roughness was found in the 7% MgO NPs subgroup, followed by the control group. Surface roughness was reduced when NPs were incorporated into the tissue conditioner, with the lowest values obtained with a minimal concentration of NPs. Furthermore, the reduced surface roughness effectively inhibited the adherence of *Candida albicans* to the tissue conditioner. A study by Al Noori et al. found that the incorporation of ZnO and MgO NPs into acrylic-based soft tissue liner decreased the adhesion of *Candida albicans* and improved resistance to fungal adherence [18]. In another study by Alwahab et al., the incorporation of ZnO NPs in tissue conditioner significantly reduced the surface roughness [12].

It was also observed in our study that the tissue conditioner incorporated with 15% ZnO NPs had the highest DIZ. Furthermore, increasing the concentration of NPs led to an increased DIZ. Therefore, based on our results, the higher the NP concentration, the greater the antifungal property.

Our findings align with those of Kanathila et al., who showed that increasing the concentration of MgO in the tissue conditioner increased the DIZ [19]. Our results also agreed with those of Homsiang et al., who showed that adding 15 wt% ZnO NPs to tissue conditioner produced an antifungal effect that lasted up to 14 days without any negative side effects [2]. Although the use of tissue conditioners is prevalent, no previous study has comparatively evaluated the effect of incorporating various concentrations of ZnO NPs and MgO NPs into tissue conditioners in terms of antifungal and physical properties.

The main limitation of the present in vitro study was that the testing was performed in a laboratory setting that did not mimic the exact oral conditions of practical applications, whereby the wet environment and simultaneous stresses were not simulated. Therefore, further in vivo investigation of the effects of different NP concentrations would be beneficial.

## Conclusions

Our results showed that the mean surface roughness was lowest in the 5% ZnO NPs subgroup and highest in the 7% MgO NPs subgroup, whereas antifungal activity was highest in the 15% ZnO NPs subgroup and lowest in the control group followed by the 3% MgO NPs subgroup. A polished surface is crucial for dental materials to prevent bacterial adhesion and plaque buildup, especially as rough surfaces can provide ideal conditions for bacteria such as *Candida albicans* to thrive. Our in vitro study showed that incorporating ZnO NPs into the tissue conditioner formulations resulted in reduced surface roughness compared to MgO NPs. These findings suggest that ZnO NPs may have potential applications in improving the surface properties and antifungal activity of tissue conditioners, which could enhance their performance
